# Avoid being trapped by your liver: ischemia-reperfusion injury in liver transplant triggers S1P-mediated NETosis

**DOI:** 10.1172/JCI167012

**Published:** 2023-02-01

**Authors:** Davide Scozzi, Andrew E. Gelman

**Affiliations:** 1 Norton Thoracic Institute, St. Joseph’s Hospital and Medical Center, Phoenix, Arizona, USA.; 2 Department of Surgery, Washington University School of Medicine, St. Louis, Missouri, USA.

## Abstract

Liver transplantation can be a life-saving treatment for end-stage hepatic disease. Unfortunately, some recipients develop ischemia-reperfusion injury (IRI) that leads to poor short- and long-term outcomes. Recent work has shown neutrophils contribute to IRI by undergoing NETosis, a form of death characterized by DNA ejection resulting in inflammatory extracellular traps. In this issue of the *JCI*, Hirao and Kojima et al. report that sphingosine-1-phosphate (S1P) expression induced by liver transplant–mediated IRI triggers NETosis. They also provide evidence that neutrophil expression of the carcinoembryonic antigen–related cell adhesion molecule-1 (CC1) long isoform inhibited NETosis by controlling S1P receptor–mediated autophagic flux. These findings suggest stimulating regulatory mechanisms that suppress NETosis could be used to prevent IRI.

## CC1-L restrains IRI and NETosis

Liver transplantation remains the only life-saving option for patients with end-stage hepatic disease. A common postoperative complication, ischemia-reperfusion injury (IRI), is the predominant driver of delayed graft function and increases the risk for poor long-term outcomes. IRI is a complex form of tissue damage triggered by the loss of arterial perfusion during the organ harvest procedure and the reconstitution of blood flow following engraftment. IRI is exacerbated by the recruitment and activation of recipient-derived inflammatory leukocytes. In the absence of observable infection, a high abundance of neutrophils within perioperative dysfunctional grafts is a canonical histological indicator of IRI. Activated neutrophils release a prodigious array of tissue-damaging molecules, including inflammatory cytokines, proteolytic enzymes, and reactive oxygen species. In this regard, experimental models of IRI repeatedly demonstrate that blocking neutrophil recruitment helps preserve perioperative graft function.

There is gathering evidence that neutrophils deliver inflammatory molecules using a potent process that promotes IRI in many transplanted organs including the liver (1). For reasons that are not yet clear, some but not all neutrophils can expel nuclear and mitochondrial DNA fibers that are bound with inflammatory cytokines, proteolytic enzymes, and histones. These extracellular DNA fibers, known as neutrophil extracellular traps (NETs), were first reported to trap and kill pathogens (2), but can cause parenchymal tissue damage in the absence of observable infection (1). Most investigation has focused on elucidating the signals that promote NETosis. These studies have identified a wide variety of stimuli, including pathogenic or tissue damage–associated molecular patterns, cytokines, sodium urate crystals, platelet activation, and immune complexes. Finding molecules or pathways that inhibit NETosis has been more challenging, since they can be camouflaged by mechanisms that simply delay apoptosis. However, there are a few notable examples, including agonists that engage the sialic acid–binding Ig-like lectin 9 or the expression of the serine protease inhibitor serpin B1, both of which are reported to inhibit *Pseudomonas aeruginosa*–induced NETosis (3, 4).

In this issue of the *JCI*, Hirao, Kojima, and colleagues report on their examination of the role of carcinoembryonic antigen–related cell adhesion molecule 1 (CC1) expression on neutrophils (5). Neutrophil CC1 expression negatively regulated NETosis and inhibited IRI in a mouse orthotopic liver-transplantation model (5). This finding is surprising, given that CC1 has been primarily described as a transmembrane biliary glycoprotein in the liver, where it plays a critical role in maintaining epithelial cell polarity, controlling insulin sensitivity and hepatic cell regeneration. *CC1* mRNA is spliced into short (S) or long (L) cytoplasmic domains in both humans and mice. On immune cells, CC1 isoforms are differentially expressed on leukocyte subsets. For example, CC1-S on regulatory CD4^+^ T cells induces suppression function, while CC1-L on T cells and neutrophils inhibits inflammatory cytokine secretion (6). Consistently, Hirao, Kojima, and others found that graft-infiltrating and bone marrow–derived neutrophils only express CC1-L, indicating that this is the isoform that intrinsically controls NETosis (5).

## S1P receptors differentially alter autophagic flux and NETosis

**Figure 1:**
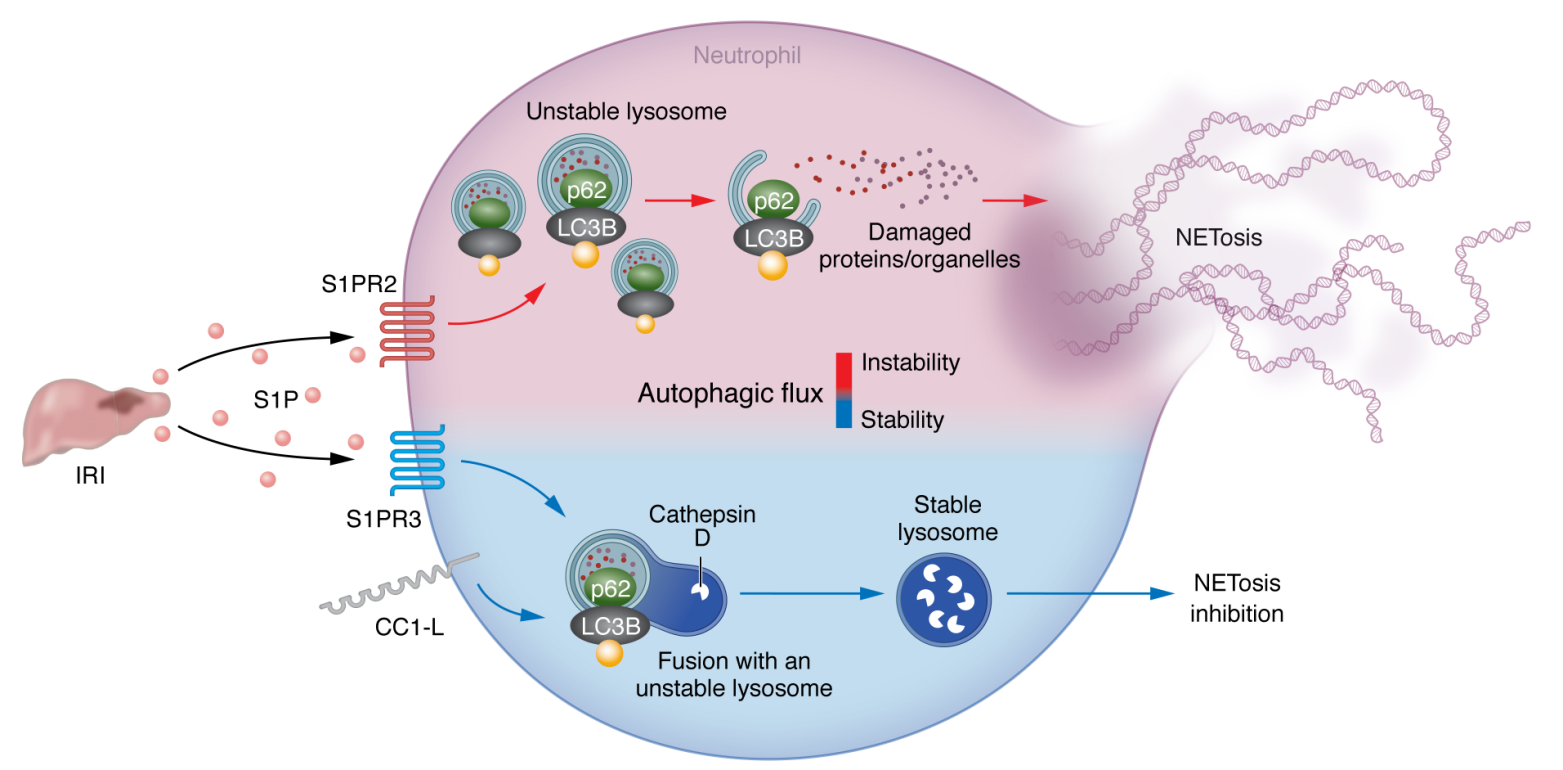
CC1-L antagonizes S1PR-mediated NETosis through inhibiting autophagic flux. IRI promotes intragraft S1P accumulation. S1P engagement with S1PR2 promotes NETosis, leading to increased autophagic flux as evidenced by p62 accumulation and lipidation of LC3B. Under high autophagic flux, leading to unstable lysosomes, NETosis becomes more likely to occur. In contrast, S1P stimulation of S1PR3 reduces autophagic flux and restrains NETosis. CC1-L antagonizes NETosis by promoting S1PR3-mediated inhibition autophagic flux as well as driving cathepsin D expression to help maintain lysosome function in response to lysosomal stress.

Noting previous work that shows CC1-deficient livers develop nonalcoholic steatohepatitis (NASH), which is exacerbated by NETosis via sphingosine-1-phosphate (S1P) expression (7), Hirao, Kojima, et al. asked whether CC1 regulates S1P-mediated NETosis. Indeed, CC1 expression on recipient neutrophils inhibited intragraft NET generation and IRI. The group also sought to identify which S1P receptors (S1PRs) controlled NETosis (Figure 1) (5). There are five S1PRs, all of which are G protein–coupled receptors that can be differentiated by a unique Gα subunit. S1PRs trigger numerous pathways, including NF-κB, phosphatidylinositol-3-kinase/AKT, and mitogen-activated protein kinase signaling pathways. The group observed that S1PR2 levels in LPS-activated neutrophils were highest in the absence of CC1 expression. In contrast, S1PR3 expression was downregulated following LPS stimulation irrespective of CC1 expression. Probing with S1PR subtype–specific antagonists revealed that S1PR2 promoted, while S1PR3 inhibited, NETosis (5). These data align with a recent report showing that S1PR2 drives NETosis in a mouse fatty acid liver model (8). However, how CC1-L alters the expression of S1PRs was not addressed by Hirai, Kojima, et al. It is interesting to note that, unlike CC1-S, CC1-L contains several immune receptor tyrosine-based inhibitory motifs (ITIM). Previous work has shown that neutrophil CC1 inhibits LPS-mediated IL-1β expression in an ITIM-dependent manner (9). IL-1β stimulates NETosis (10), which raises the possibility that CC1 could inhibit autocrine factor expression that drives NETosis.

Autophagy is generally viewed as a cellular survival mechanism that operates by shuttling protein aggregates and damaged organelles to lysosomes in response to nutrient starvation, pathogen infection, or oxidative stress. Autophagic activity, also called “autophagic flux,” can be assessed by the accumulation of p62, which marks protein complexes and organelles for destruction, and the lipidation of microtubule-associated protein light chain 3β (LC3B-II), which is required for autophagosome membrane generation. Reports differ as to whether autophagy is required for NETosis, which is likely due to how NETosis is induced and the resulting magnitude of autophagic flux (11). Additionally, the role of S1P signaling in autophagy appears contradictory, as S1P can promote and prevent autophagy, possibly due to differential activity and expression of S1PRs (12, 13). Work by Hirao, Kojima, and colleagues appears to bear this out. Through using specific inhibitors to S1PR2 and -3, the authors’ provide data that indicate that S1PR2 stimulation promotes p62 expression and lipidated LC3B formation while S1PR3 engagement inhibits autophagic flux by preventing LC3B lipidation. CC1 also inhibited autophagy following the induction of lysosomal stress. Treatment of neutrophils with bafilomycin A, which prevents lysosomal acidification by inhibiting the proton-pumping capability of the vacuolar ATPase complex, reduced autophagic flux and NETosis in WT but not in *CC1*-KO neutrophils following S1PR2 blockade, suggesting that CC1 promotes S1PR3 activity. Further analysis indicated that CC1 increased lysosomal stability, as *CC1*-KO neutrophils failed to maintain expression of cathepsin D, a proteolytic enzyme that digests lysosomal cargo. In line with observations in mice, the group also observed that CC1-L levels from human liver transplant biopsy tissue inversely correlated with the lysosomal protease cathepsin G. Moreover, a low CC1-L–to–cathepsin G ratio was associated with enhanced graft damage and increased evidence of NETosis (5). Although it remains unknown whether cathepsins regulate NETosis, cathepsin D deletion from mouse neutrophils has been shown to prolong innate immune responses by delaying apoptosis (14). Moreover, neutrophil lysosomal instability (15) and S1PR2-mediated inhibition of apoptosis (8) are both reported to stimulate NETosis.

## Targeting NETosis as a strategy for preventing IRI

Despite convincing clinical evidence that NETosis contributes to IRI-mediated graft dysfunction and tissue damage, NET-targeted therapies have yet to be rigorously evaluated in the transplant clinic. One potential therapy involves degrading NETs. Aerosolized forms of recombinant human deoxyribonuclease (DNase) improve lung function in patients with cystic fibrosis (16), whose airway mucus is highly enriched for NETs. Intravascular injection of DNAse may have the added benefit of improving organ perfusion by degrading NETs that plug the capillary lumen (17). However, work in experimental models has shown DNAse generates NET fragments that prevent allograft tolerance, suggesting that inhibiting NETosis may be preferable to pharmacological NET degradation (18). mTORC1s, which are commonly used to inhibit T cell activation in liver transplant recipients (19), are reported to prevent human NETosis in vitro (20) and blunt warm liver IRI in mice (21). Additionally, the antidiabetic drug metformin has been shown to prevent NETosis in prediabetic patients (22). Perhaps more compelling, the finding that both mTORC1 inhibitors rapamycin and metformin inhibit the activity of the vacuolar ATPase complex (23, 24) underscores the observations made with bafilomycin A in Hirao, Kojima, et al. Nevertheless, targeting NETosis in the treatment of IRI may pose risks to an already immunocompromised transplant recipient. Because defects in NETosis increase vulnerability to bacterial and fungal infection (25), the development of such therapies should carefully consider effects on pathogen surveillance. We eagerly await future work.

## Version 1 02/01/2023

Electronic publication
